# Phenylalanine and tyrosine levels are rate-limiting factors in production of health promoting metabolites in *Vitis vinifera* cv. Gamay Red cell suspension

**DOI:** 10.3389/fpls.2015.00538

**Published:** 2015-07-16

**Authors:** Neta Manela, Moran Oliva, Rinat Ovadia, Noga Sikron-Persi, Biruk Ayenew, Aaron Fait, Gad Galili, Avichai Perl, David Weiss, Michal Oren-Shamir

**Affiliations:** ^1^Agricultural Research OrganizationBet-Dagan, Israel; ^2^Institute of Plant Sciences and Genetics in Agriculture, Faculty of Agricultural, Food, and Environmental Quality Sciences, The Hebrew University of JerusalemRehovot, Israel; ^3^Department of Plant Science, The Weizmann Institute of ScienceRehovot, Israel; ^4^Ben-Gurion University of the Negev, Jacob Blaustein Institutes for Desert ResearchBeersheba, Israel

**Keywords:** phenylalanine, tyrosine, phenylpropanoids, DAHPS, *Vitis vinifera*, grape cell suspension

## Abstract

Environmental stresses such as high light intensity and temperature cause induction of the shikimate pathway, aromatic amino acids (AAA) pathways, and of pathways downstream from AAAs. The induction leads to production of specialized metabolites that protect the cells from oxidative damage. The regulation of the diverse AAA derived pathways is still not well understood. To gain insight on that regulation, we increased AAA production in red grape *Vitis vinifera* cv. Gamay Red cell suspension, without inducing external stress on the cells, and characterized the metabolic effect of this induction. Increased AAA production was achieved by expressing a feedback-insensitive bacterial form of 3-deoxy- D-arabino-heptulosonate 7-phosphate synthase enzyme (AroG^*^) of the shikimate pathway under a constitutive promoter. The presence of AroG^*^ protein led to elevated levels of primary metabolites in the shikimate and AAA pathways including phenylalanine and tyrosine, and to a dramatic increase in phenylpropanoids. The AroG^*^ transformed lines accumulated up to 20 and 150 fold higher levels of resveratrol and dihydroquercetin, respectively. Quercetin, formed from dihydroquercetin, and resveratrol, are health promoting metabolites that are induced due to environmental stresses. Testing the expression level of key genes along the stilbenoids, benzenoids, and phenylpropanoid pathways showed that transcription was not affected by AroG^*^. This suggests that concentrations of AAAs, and of phenylalanine in particular, are rate-limiting in production of these metabolites. In contrast, increased phenylalanine production did not lead to elevated concentrations of anthocyanins, even though they are also phenylpropanoid metabolites. This suggests a control mechanism of this pathway that is independent of AAA concentration. Interestingly, total anthocyanin concentrations were slightly lower in AroG^*^ cells, and the relative frequencies of the different anthocyanins changed as well.

## Introduction

Phenylalanine and tyrosine are key players in the production of the large and diverse family of phenylpropanoid specialized metabolites. Phenylpropanoids play a central role in the adaptation of plants to changing environmental conditions and in their defense against pathogens (Dixon and Paiva, 1995). In addition, these metabolites also participate in plant development and reproduction processes (Vogt, 2010). Both amino acids are products of the shikimate and aromatic amino acids (AAA) pathways and are the source for phenylpropanoids, linking primary to specialized metabolic pathways. The phenylpropanoid pathways include the flavonoid and anthocyanin, benzenoid, stilbenoid, and lignin pathways. In contrast to the knowledge on the regulation of the shikimate and AAA pathways (Bentley, 1990; Tzin and Galili, 2010; Maeda and Dudareva, 2012), the inter-regulation between these pathways and the diverse phenylpropanoid pathways is poorly understood. One reason for this is the diversity of phenylpropanoids across plants, plant organs and growth conditions.

Exposure of plants to various stresses induces the expression of genes along the shikimate, AAA and the different phenylpropanoid pathways (Tzin and Galili, 2010; Less and Galili, 2008). In some cases the shikimate and AAA pathways are induced directly. For example, oligogalacturonides released due to pathogen infection, induce genes along the shikimate and AAA pathways, as well as genes along the phenylpropanoid pathways (Ferrari et al., 2007). Several stress responses cause direct induction of one or several of the phenylpropanoid pathways, and this in turn may affect the shikimate and AAA pathways due to the increased catabolism of their products, phenylalanine (Phe) and tyrosine (Tyr). The phenylpropanoid pathways downstream of the AAA pathway, such as flavonoid, anthocyanin, and stilbenoid pathways, are often induced due to environmental stresses such as low temperature or high light intensity and in particular UV light (Chalker-Scott, 1999; Winkel-Shirley, 2002). The different phenylpropanoid pathways resulting from the catabolism of Phe and Tyr are differentially induced, depending on the plant, developmental, and environmental conditions, and both biotic and abiotic stresses (Castellarin et al., 2007; Naoumkina et al., 2010; Dai et al., 2013; Degu et al., 2014).

Grape berries accumulate high concentrations of diverse phenylpropanoids including flavonoids, anthocyanins, benzenoids, and stilbenoids (Adams, 2006). Various studies showed that consumption of grape products can reduce the incidence of chronic illnesses, such as cancer, cardiovascular diseases, ischemic stroke, neurodegenerative disorders, and aging (Iriti and Faoro, 2009). Grapevines utilize primary metabolites to synthesize three main classes of bioactive phytochemicals: phenylpropanoids, isoprenoids, and alkaloids. Within the phenylpropanoids, many compounds accumulating in red grapes have health promoting characteristics and were shown to prevent development of chronic diseases such as cancer, diabetes, and obesity (Howitz and Sinclair, 2008; Martin et al., 2011). The best known phenylpropanoids in grapes with health promoting effects are the flavonol quercetin and the stilbenoid resveratrol (Baur and Sinclair, 2006; Westphal et al., 2007). In addition, anthocyanins, responsible for the red coloration of grape berries, were also proven as health promoting metabolites that protect cancer–prone mice when added at high levels to their diet (Butelli et al., 2008). These metabolites, rich in wine, are thought to be responsible for the “French-paradox” (Reisch et al., 2012), a term describing the observation that the French have relatively low cardiovascular diseases despite a high consumption of saturated fats.

Resveratrol production in grapes is induced due to biotic or abiotic stresses. Stilbene synthase, whose activity leads to the production of resveratrol, has been shown to be induced due to *Botrytis cinerea* infection, treatment with aluminum chloride and UV irradiation (Borie et al., 2004). The UV-C induction of resveratrol production in grape berries was shown to be dependent on the developmental stage of the berries, with the most significant induction occurring before veraison (Wang et al., 2015).

Plant cell suspensions provide an attractive alternative to the whole plant for molecular and biochemical studies of metabolic pathways (Ferri et al., 2009; Mewis et al., 2011; Mustafa et al., 2011). Cell cultures of grape berries developed from various *Vitis vinifera* red and green cultivars have been widely used in the research community (Mustafa et al., 2011). *Vitis vinifera* cv. Gamay cell suspensions derived from disk of the ripening red berries were developed by several research groups and used widely in studies on anthocyanin and stilbenoid biosynthesis since the 1990's (Cormier et al., 1990). Their biology, growth rate, anthocyanin accumulation (Sinilal et al., 2011), and induction of stilbenoids (Kiselev, 2011) are well documented. Previous studies showed that the metabolic profile of phenylpropanoids in grape cell cultures is dependent on the growth conditions, and varies between different grape varieties (Ferri et al., 2009; Mewis et al., 2011). These findings suggest that further understanding of the dynamics and regulation of AAA-derived secondary metabolites may improve their utilization.

To gain insight on the inter-regulation between the shikimate and AAA pathways and the phenylpropanoid pathways in red grapes, we increased the production of Phe and Tyr in red grape *Vitis vinifera* cv. Gamay Red cell suspension without inducing external stress on the cells, and characterized the metabolic effects of this induction. Increased AAA production was achieved by expressing a feedback-insensitive bacterial form of 3-deoxy-D-arabino-heptulosonate 7-phosphate synthase enzyme (AroG^*^) of the shikimate pathway under a constitutive promoter. Our results suggest that increased internal concentrations of Phe and Tyr caused a significant increase in several specialized metabolites representing different phenylpropanoid pathways, without inducing the metabolic genes along these pathways.

## Materials and methods

### Plant material

The grape (*Vitis vinifera*) cell suspension originated from disk (1-2 mm) of berries at veraison (1 cm) of the variety Gamay Red, as described in Gollop et al. (2002). Liquid wt cultures were grown on Gamborg salts (pH 5.9) with B5 vitamins (Duchefa, The Netherlands), supplemented with 250 mg/l casein hydrosylaze, 100 mg/L myo-inosytol, 0.2 mg l-1 kinetin and 0.1 mg l-1 NAA, 20 g l-1 sucrose and 0.8% (w/v) agar (Gollop et al., 2002). Cells were cultured in liquid medium with the same nutrient and hormone composition and maintained at 25 ± 1°C with constant shaking at ±100 rpm under constant light conditions (25 μmol m^−2^s^−1^). A 50-ml stock of suspended cells was maintained by sub-culturing 2.5 gr of cell to fresh medium once a week.

### Generation of stable transgenic Gamay Red cell lines

The procedure for transformation of *Vitis vinifera* Gamay Red was modified from that described by Gollop et al. (2002). Sterile wt Gamay-Red grape cells grown in liquid medium were used for *Agrobacterium tumefaciens* mediated transformation at day 7 after reculturing. *A. tumefaciens* bacterial cultures were grown over-night at 28°C, 2 days before transformation on LB medium supplemented with 100 μM acetosyringone with the appropriate selection (25 μg/L Rifampicin and 100 μg/L Spectomycin which is the selectable marker on the plasmid of interest). A day before transformation, *A. tumefaciens* was recultured on LB supplemented with 500 μM acetosyringone, 1 μM MgSO_4_ and the appropriate plasmid selection and grown to O.D_600_ 1. At the transformation day, agro was pelleted (5000 rpm) for 10 min at room temperature, washed twice in the grape cell culture medium and resuspended to O.D_600_ 0.4 in grape cell growth medium to a final volume of 30–40 ml supplemented with 500 μM acetosyringone, 10 μM MgSO_4_, (without selection) and grown at 28°C for 3–4 h.

Grape cells were treated only shortly before co-cultivation with the agro cells to avoid stressful conditions. Grape cells were centrifuged at 830 rpm (100G) for 5 min at RT. Final volume of fresh grape cell pellet without medium was 6-7 ml. Upon inoculation, agro cells were transferred to 250 ml flasks, supplemented with 100 mg/L DTT, and the grape cells. Flasks were agitated in a shaker at RT at 70 rpm for 30 min, which were followed by centrifugation in 830 rpm, 5 min, to take all agro medium out. Volume of grape cells was measured.

For co-cultivation, grape cells were spread in a condensed manner (1.5 ml cell pellet per plate) on solid grape medium plates supplemented with 100 mg/L DTT and 100 μM acetosyringone and incubated in the in RT and dark conditions for 2–3 days. Before grape cells were spread on co-cultivation plates, the plates were covered with sterile 325 P cellulose film (cellophane paper) 80 mm diameter (A.A Packaging limited, UK) to allow easy handling of cells collection or medium replacement. Following co-cultivation without selection, grape cells were collected, washed twice in liquid medium and transferred to liquid medium flasks with selection against agrobacteria (liquid grape medium + 250 mg/L cefatoxime + 100 mg/L DTT). Fresh cell volume was 20% of final volume of the grape liquid medium. Selection against agro cells was performed in liquid grape medium supplemented with 100 mg/L DTT which was replaced every day, for 3–5 days. At day 3–5 from end of co-cultivation grape cells were pelleted and spread over solid selection medium, covered with cellophane paper (1.5 ml for one plate) which contained both selection against agro and antibiotics used as a selectable marker for successful growth of transgenic cells (50 μg/L Kanamycin) and 100 mg/L DTT. Plates were incubated in RT and dark conditions. Solid medium replacement was performed once a week. Four to eight weeks later colonies were developed and separately transferred to new selection medium for further growth. Molecular analysis of transgenic cells from several different colonies was performed for validation of transgenic enzyme expression.

### Plasmid construction and PCR amplification

The feedback-insensitive chimeric *AroG*_175_ construct (Tzin et al., 2012) used for the grape cell suspension transformation contains a 35S CaMV promoter, a plastid transit peptide (TP) fused in frame to 5′ end of the truncated *AroG* feedback-insensitive bacterial gene (AroG^*^), hemagglutinin (HA) epitope tag fused in frame at the 3′ of the AroG^*^ gene and a terminator. AroG_175_ contained a point mutation in position 524 bp (Leu175Glu). For the generation of controls, wt cell suspension was transformed with the same plasmid used for transformation, containing only the kanamycin resistance cassette. The following PCR primers were used:

Forward 5′-GTGCACCGCGAACAGGCATCAGGGCTT-3′

Reverse 5′- AAGCCCTGATGCCTGTTCGCGGTGCAC-3′

### Immunoblot analysis

Immunoblots were performed as previously described (Stepansky and Galili, 2003), using monoclonal anti-HA antibodies (Sigma-Aldrich) with the following modification: Total protein was extracted from samples of 100 mg (FW) cell culture and grounded in liquid nitrogen containing 20 mg Polyvinylpolypyrrolidone (PVPP) and 200 μl potassium phosphate buffer (25 mM), and freshly added protease inhibitors (Complete, ROCHE) and 1 mM PMSF. Soluble protein extracts were separated from cell precipitates by centrifugation at 14,000 rpm for 10 min, and boiled in protein sample buffer. Ponceau staining and de-staining were performed using Ponceau dye (Sigma-Aldrich) according to the manufacturer's instructions. The intensity ratio between the four AroG^*^ lines was determined using ImageJ (image processing and analysis in Java) software, version.

### Anthocyanin extraction and characterization

Cell pellets from 2 ml liquid culture were collected and grounded in liquid nitrogen. Pigments were extracted with 2 ml of cold methanol:water:acetic acid (11:5:1) according to Markham and Ofman (1993). Extracts were centrifuged for 10 min at 800 rpm. Supernatants were concentrated to 0.5 ml, hydrolyzed by boiling with equal volumes of methanol and 2N HCl for 1 h and passed through a 0.45–L m polyvinylidene difluoride filter (Nalgene, Waltham, MA). Anthocyanin composition was determined using high-performance liquid chromatography (HPLC) (Shimatzu, Kyoto, Japan) equipped with an LC-10AT pump, an SCL-10A controller and an SPD-M10AVP photodiode array detector, as described in Oliva et al. (2015).

### GC-MS extraction, derivatization and profiling of metabolic extracts

Fresh Gamay cells from day 9 were washed twice with distilled water at 4°C and lyophilized (Christ LMC-1 Gamma 1–20, Osterode, Germany). Metabolites were extracted from 100 mg dried grape extract powder, in chloroform:methanol:water at a ratio of 1:1:2.5. 0.2 mg/ml ribitol was added as an internal standard. The mixture was vortexed for 10 min and sonicated for 10 min at 7–8°C. Samples were centrifuged for 2 min at 14000 rpm, after addition of 300 μl DDW and 300 μl chloroform. GC-MS derivatization and data processing was performed according to Hochberg et al. (2013).

### Real time PCR

Total RNA was isolated from the grape cells at different times by ZR Plant RNA MiniprepTM (ZYMO, USA) followed by DNase treatment (Qiagen, Germany). First-strand cDNA was synthesized from 2.5 μg RNA using RevertAidTM M-MuLV reverse transcriptase (Fermentas, Canada). Realtime amplification was performed using Fast SYBR Green (Applied Biosystemms, USA) master mix in a ABI StepOnePlus (Applied Biosystemms, USA) using a comparative (ΔΔ*C_t_*) study. Keeping the total reaction volume as 20 μl, 40 amplification cycles were carried out consisting of denaturation at 95°C for 15 s, annealing at 60°C for 15 s and extension at 72°C for 20 s. Relative expression was determined by normalizing the values to that of ubiquitin. PCR efficiency was calculated from seven-fold serial dilutions of plasmids for each primer pair in triplicates (Table 1). Primers for real-time amplification were designed based on the coding sequences of Vitis species available at NCBI.

**Table 1:** **Primers used for quantitative real-time PCR of genes of the phenylpropanoid biosynthetic pathways**.

**Gene name**			**Sequence 5′–3′**
Phenylalanine ammonia lyase	Pal	Sense	5′- TCC TCC AAA TGC CTC AAA TC -3′
		Anti	5′- GCT GAG CAA CAC AAC CAA GA -3′
Chalcone synthase	Chs	Sense	5′- GAG ACC TGG TCC AAA TCC AA -3′
		Anti	5′- CTA GTG CAT GCG TGC TGT TT- 3′
Stilbene synthase	Sts	Sense	5′- TCC GAA GAT GCT TTG GAC TC -3′
		Anti	5′- GTG GTC GTT CAA TCG AGA CA -3′
Anthocyanin synthase	Ans	Sense	5′- TGA GAG CTT GTC CAG CAG TG -3′
		Anti	5′- GGC CCT TCA TCC TTC TTC TC -3′
Flavanoid 3′ hydroxylase	F3′h	Sense	5′- GGT TAA CAG GTG CAC CAT CC -3′
		Anti	5′- TAG AAT TCA GGC CCA ACA GG -3′
Dihydroflavonol 4-reductase	Dfr	Sense	5′- ATG GAA TGT CAT GCG TGC TA -3′
		Anti	5′- GAA GTG GGT GCG TAC CTG AT -3′
Flavanoid 3′, 5′ hydroxylase	F3′5′h	Sense	5′- CGT GTG CTC CTC CAT CAT -3′
		Anti	5′- CAT GAC CAG TGC TGG GTA CTT -3′
Ubiquitin	Ub	Sense	5′- AAC CTC CAA TCC AGT CAT CTA C -3′
		Anti	5′- GTG GTA TTA TTG AGC CAT CCT T-3′

### Statistical analysis

Statistical significance analysis by One-Way ANOVA test followed by Dunnett's *post–hoc* test in which *P* ≤ 0.05, using JMP software (NC, USA). Standard error of fold change results was calculated by dividing each repeat to the mean of the 3 replicates for each variant.

## Results

### Generation of transgenic *Vitis vinifera* cv. Gamay Red cell culture expressing the bacterial AroG^*^ gene

**Figure 1:**
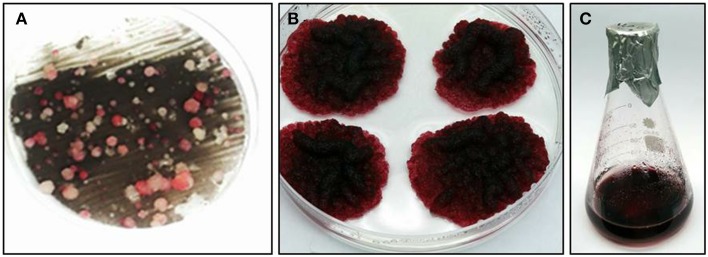
**Transformation of transgenic** ***Vitis vinifera*** **cv. Gamay cell culture**. **(A)** kanamycin resistant cell culture colonies, from which the four AroG^*^ lines were chosen. **(B)** Typical AroG^*^ cell culture colonies, **(C)** Growth of the culture in liquid medium.

*Vitis vinifera* cv. Gamay cell suspension, derived from disk of ripening red berries, were chosen for this study due to the accumulated knowledge on their anthocyanins and stilbenoids biosynthesis and a published transformation protocol for this culture (Gollop et al., 2002). Gamay cell suspension was transformed with a feedback-insensitive bacterial DAHPS (AroG^*^) gene (see Materials and Methods), encoding for the first enzyme of the shikimate pathway, under the control of the 35S promoter (Tzin et al., 2012). Cells were grown on solid medium to isolate colonies from different transformation events. Four independently transformed lines were selected for metabolic and transcriptomic analysis. The selected lines developed relatively large colonies with a dark red pigmentation (Figure 1), suggesting rapid growth rate and high anthocyanin pigmentation. The selected four AroG^*^ lines and two control lines, transformed with the same plasmids not including the AroG^*^ construct, have been stable for more than 3 years both in solid and liquid cultures, with unaltered rates of growth and pigment accumulation.

**Figure 2:**
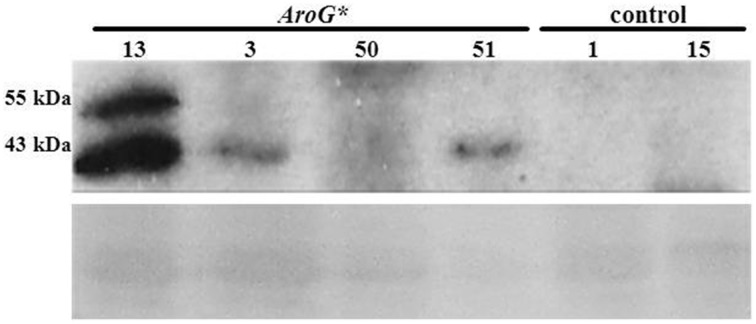
Accumulation of AroG^*^ protein in four transgenic Gamay cell culture lines. Immunoblot analysis was performed on protein extracts of the four AroG^*^ lines and two controls, using anti-HA antibody (1:1000). The major protein band at 43 kDa and the additional band slightly above it represent the mature and the unprocessed AroG^*^ protein, respectively. Ponceau staining of the gel indicates similar protein loading. The relative intensity of the 43 KDa band was 10.9:3.7:1:2.5 in lines 13, 3, 50, and 51 respectively.

Analysis of AroG^*^ protein accumulation in the transgenic Gamay Red lines, revealed two polypeptide bands, the major, lower band corresponding in size to the mature AroG^*^ polypeptide (42.5 kDa) and the upper band to the unprocessed AroG^*^ containing the plastid transit peptide (Figure 2). The difference in the intensities of the two bands indicates that most of the AroG^*^ protein has been processed and translocated into the plastids. Of the four lines, line 13 accumulated the highest levels of AroG^*^ protein.

**Figure 3:**
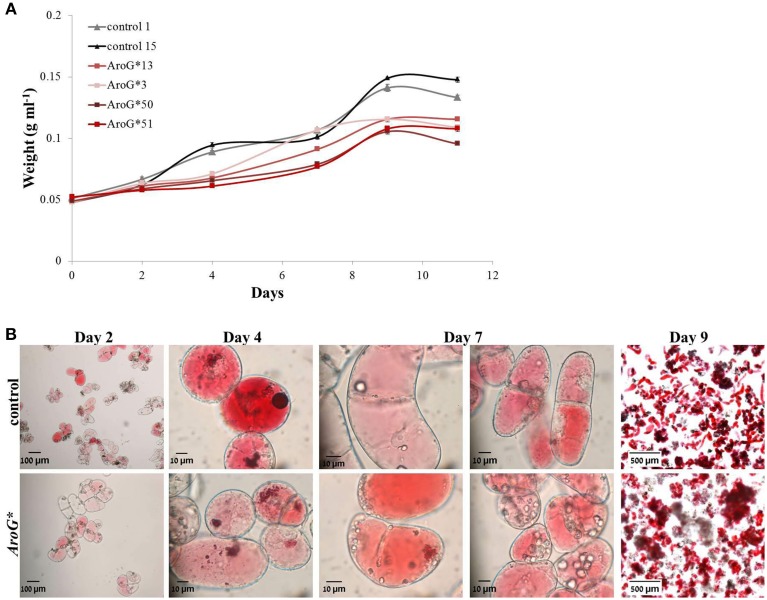
**Growth and morphology of** AroG^*^ cell lines. (A) The growth curve of the cultures was determined by the increase in fresh weight from the day of reculturing (day 0) until beginning of cell death. **(B)** Microscopic photographs (taken by a Leica MZ FLII microscope) of line 13 and control cells during their growth. Bars on top of the histograms indicate standard errors (*n* = 3).

The four chosen lines accumulating AroG^*^ protein, grew both on solid and liquid media (Figures 1B,C). The growth rate of the AroG^*^ lines in a liquid media was slightly slower than control lines containing an empty vector (Figure 3A). AroG^*^ cells looked identical to those of controls, with a slightly lighter color in two of the lines, 50 and 51. Both in controls and in AroG^*^ lines, the cells were relatively small and colorless after reculturing into fresh medium. Anthocyanin accumulation began parallel to enlargement of the cell vacuoles and before division (Figure 3B, Day 2). Since the cultures were not synchronized, a variety of cells at different developmental stages are present at each given time. The main visible difference between the AroG^*^ lines and controls was that the cells in the AroG^*^ cultures grew in larger clumps in the liquid media, in comparison to control lines (Figure 3B, Day 9).

**Figure 4:**
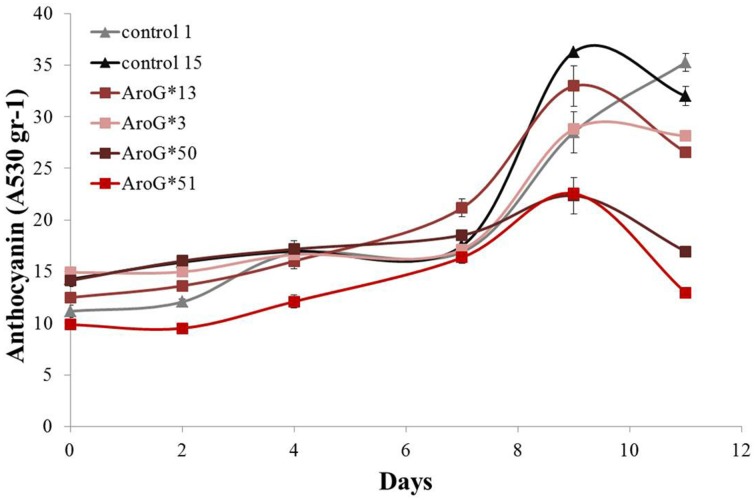
**Effect of** AroG^*^ transformation on total anthocyanin accumulation in the Gamay cell cultures. The increase in total anthocyanin concentration was determined as the increase in OD absorption at 530 nm. Bars indicate standard errors (*n* = 3).

The rate of anthocyanin accumulation varied between the four lines: lines 13 and 3 accumulated anthocyanins at a similar rate as controls, while in lines 50 and 51 accumulation was slower (Figure 4) and the cultures had a lighter color. Clearly high AroG^*^ abundance had no direct significant effect on total anthocyanin accumulation, since both lines with the higher abundance had similar pigment concentrations as controls. The peak of anthocyanin accumulation in the cells was at day 9 (Figure 4), similar to the growth peak (Figure 3A).

### Effect of AroG^*^ accumulation on the metabolic profile of Gamay Red cells

**Figure 5:**
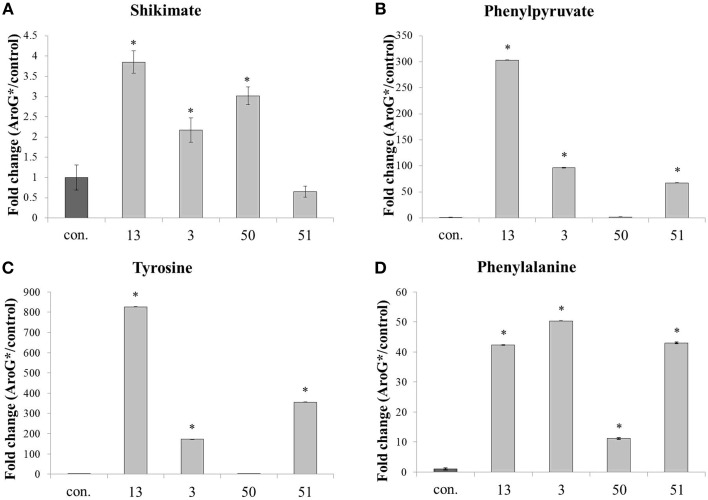
**Effect of** AroG^*^ **transformation on the levels of shikimate and AAA metabolites**. Levels of metabolites [**(A)** shikimate, **(B)** phenylpyruvate, **(C)** tyrosine, and **(D)** phenylalanine] are presented as fold increase for each AroG^*^ line in comparison with the average of the two control lines. Samples were collected at day 9 of growth. Bars on top of the histograms indicate standard errors (In the control *n* = 6, including 3 samples from each of the two lines, while in the AroG^*^ lines *n* = 3). Statistical significance was analyzed by One-Way ANOVA, followed by Dunnett's *post–hoc* test. Asterisks indicate *P* ≤ 0.05.

A metabolomics comparison was performed between the four AroG^*^ transgenic lines and two control lines. Samples from the six lines were subjected to GC-MS and HPLC analysis to detect metabolites that differentially accumulate in the transgenic lines. Primary metabolites leading to formation of AAAs, as well as two AAAs, Phe, and Tyr, increased significantly in the AroG^*^ lines (Figure 5). Shikimate, a product of the shikimate pathway, increased 2-3 fold in three of the four transgenic lines, with the highest level observed in the line with the highest abundance of AroG^*^ protein (line 13). Phenylpyruvate, an intermediate of the AAA pathway, also increased in three of the four transgenic lines, with the highest level of over 240 fold increase in line 13. AroG^*^ abundance caused significant increase both in Phe and Tyr, while Trp was not detected in the cells. The initial concentration of Tyr in controls was about 10% that of Phe (results not shown). However, this ratio changes dramatically in the AroG^*^ lines, with a much higher increase in Tyr concentrations in comparison to Phe (800 and 40 fold increase, respectively, in line 13). The transformed lines show large variation in the concentrations of these metabolites. However, line 13, with the highest abundance of AroG^*^, was consistently high in all four metabolites.

**Figure 6:**
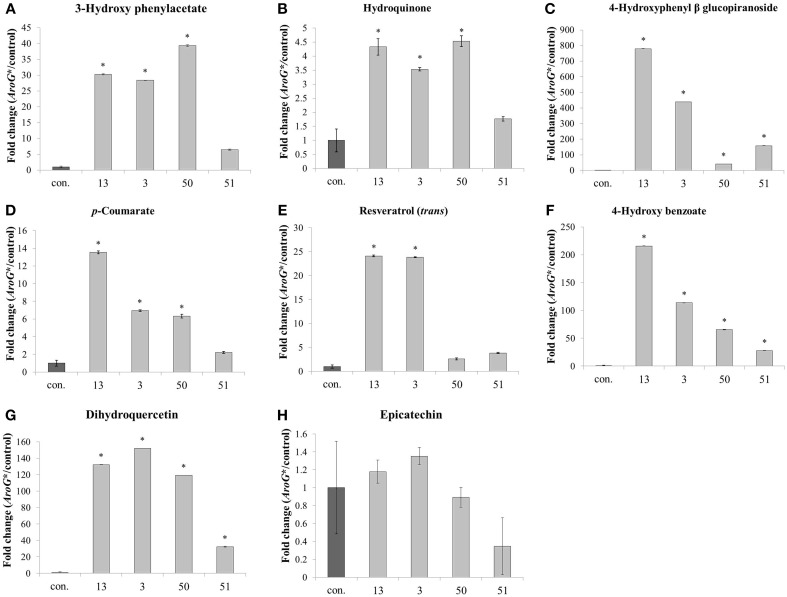
**Effect of** AroG^*^ transformation on the levels of AAA-derived specialized metabolites. Levels of metabolites [**(A)** 3-hydroxy phenylacetate, **(B)** hydroquinone, **(C)** 4-hydroxyphenyl β glucopiranoside, **(D)** *p*-coumarate, **(E)** resveratrol, **(F)** 4-hydroxy benzoate, **(G)** dihydroquercetin, and **(H)** epicatechin] are presented as fold change of AroG^*^ line in comparison with the control line. Bars on top of the histograms indicate standard errors (In the control *n* = 6, including 3 samples from two lines, while in the AroG^*^ lines, *n* = 3). Statistical significance was analyzed by One-Way ANOVA, followed by Dunnett's *post-hoc* test. Asterisks indicate *P* ≤ 0.05.

In addition to the increased levels of Phe and Tyr, AroG^*^ cell lines also accumulated high levels of specialized metabolites derived mainly from Phe and also possibly from Tyr (Figure 6). These metabolites represent different pathways branching from these AAAs, including flavonoids, stilbenoids and benzenoids. Of the eight non-pigment AAA-derived specialized metabolites detected in the Gamay Red cell culture, epicatechin was the only metabolite that did not increase in the transgenic lines. This may be due to the fact that epicatechin is produced from anthocyanins, and anthocyanin levels remained unchanged in lines 3 and 13, and decreased in lines 50 and 51 (Figure 4). All other non-pigmented metabolites increased significantly in the two lines with the highest AroG^*^ protein abundance, lines 13 and 3. Line 50 showed significant increase in six of the metabolites, while line 51 showed significant increase in three of the phenylpropanoid specialized metabolites (Figure 6).

**Figure 7:**
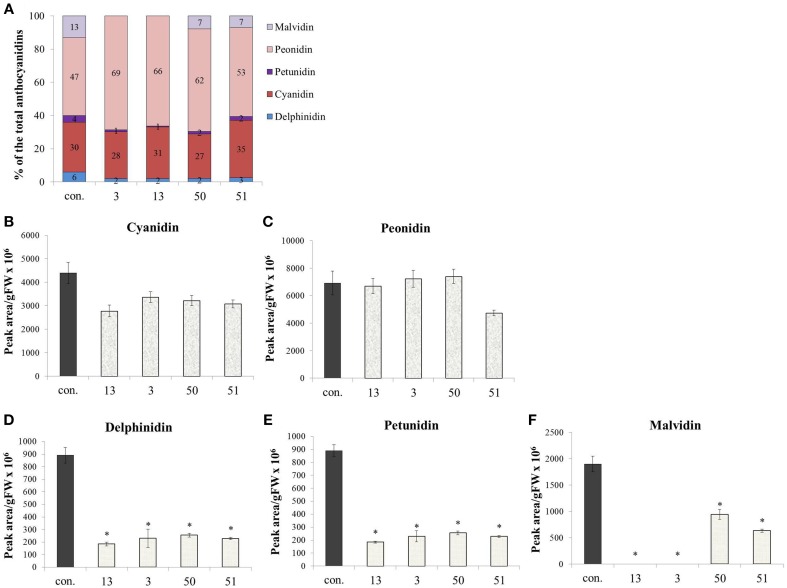
Effect of AroG^*^ abundance on the cell cultures' anthocyanin aglycon composition. **(A)** The distribution between the five anthocyanin aglycons in the transgenic lines. **(B–F)** The concentration of the five aglycons: **(B,C)** The two major aglycons, cyaniding and peonidin, **(D–F)** the three minor components. Bars on top of the histograms indicate standard errors (In the control *n* = 6, including 3 samples from two lines, while in the AroG^*^ lines, *n* = 3). Statistical significance was analyzed by One-Way ANOVA, followed by Dunnett's post hoc test. Asterisks indicate *P* ≤ 0.05.

Anthocyanins, also derived from Phe, did not increase in their concentration due to increased Phe production in the AroG^*^ lines, as noted in Figure 4. However, a closer look at the ratio between the anthocyanin aglycones (anthocyanidins) in the grape cells revealed a substantial difference between AroG^*^ lines and controls. There are five anthocyanidins responsible for the pigmentation of the Gamay cell culture. Peonidin and cyanidin, derived from dihydroquercetin, comprise close to 80% of cell suspension anthocyanidins, while delphinidin, malvidin, and petunidin, derived from dihydromyricetin, comprise around 20% of the total pigment concentration in the cells (Figure 7A). Peonidin and cyanidin did not vary significantly between AroG^*^ lines and controls (Figures 7B,C). However, the three dihydromyrictin-derived aglycons accumulated significantly lower levels in all AroG^*^ lines in comparison to controls (Figures 7D–F). Malvidin was not detected in the strongest AroG^*^ lines, 13 and 3 (Figure 7F). This suggests a different regulatory effect in the transformed cells between the two enzymes separating these two branches of the anthocyanin biosynthetic pathway, flavonoid 3′-hydroxylase (F3′H), and flavonoid 3′5′-hydroxylase (F3′5′H).

### Is the increase in specialized metabolites in AroG^*^ lines due to induction of biosynthetic genes?

Increased production of Phe and Tyr resulted in dramatic accumulation of specialized metabolites in several pathways derived from Phe and Tyr (Figures 6, 7). To test if this effect was a result of induction of genes along the specialized metabolic pathways or just due to the increase in substrate availability, a quantitative real time PCR analysis was performed on seven key genes, including phenylalanine ammonia lyase (PAL), the first enzyme along the phenylpropanoid pathway, stilbene synthase (STS), the key enzyme in production of stilbenoids and chalcone synthase (CHS) the first enzyme in production of flavonoids (**Figure 9**). The transcripts' levels were determined for cells at three time points of growth: day 6, when the accumulation of anthocyanin, a representative of the AAA derived metabolites, begins, day 7, and day 9, at the peak of pigmentation and weight (Figures 3A, 4). In three of the four AroG^*^ lines, including line 13 with the highest AroG^*^ protein abundance, there was no significant induction of all seven genes throughout the cell culture growth, suggesting that Phe and possibly also Tyr were the rate limiting factors in production of downstream specialized metabolites. In contrast, in line 3, most of the seven genes tested were induced in all three time points tested, probably due to the specific position of the inserted gene in this line. Despite the high levels of transcripts in line 3, this line was not outstanding in the levels of the specialized metabolites. In fact, line 13, with high AroG^*^ protein abundance but no significant gene induction had either equal or higher levels of specialized metabolites in comparison to line 3 (Figure 6), suggesting that substrate availability and not the levels of biosynthetic enzymes, is the limiting factor in the production of these metabolites.

## Discussion

**Figure 8:**
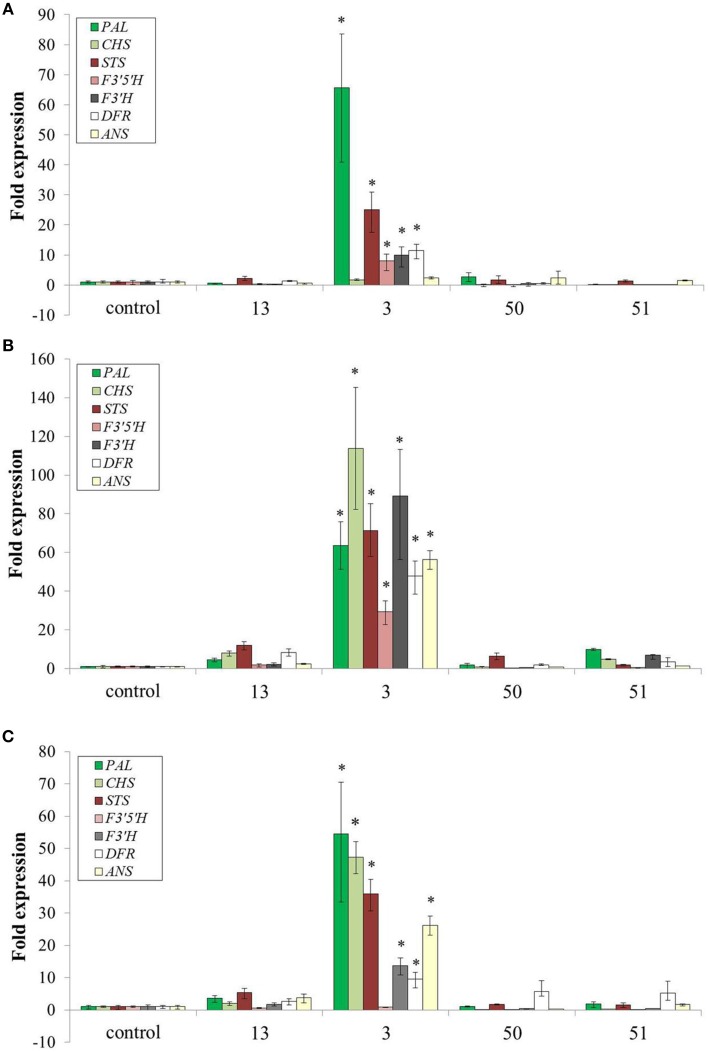
Effect of AroG^*^ protein on the transcript levels of key genes related to specialized metabolites. **(A)** day 6, **(B)** day 8, and **(C)** day 9. Bars on top of the histograms indicate standard errors (*n* = 3). One of the two control lines was included (line 15). Statistical significance was analyzed by One-Way ANOVA, followed by Dunnett's post hoc test. Asterisks indicate *P* ≤ 0.05.

Transformation of grape cell culture with AroG^*^ mimicked a stress response in the cells, while growing at optimal nonstressful conditions: The AroG^*^ grape cells accumulated high concentrations of phenylpropanoids (Figure 6), including representatives from several pathways branching from Phe and Tyr. Metabolites from the stilbenoid, flavonoid, and benzenoid pathways were overproduced in these cells, with a significant and high fold increase in comparison to controls. The end result of increased phenylpropanoids was in fact similar to cells under stressful conditions. However, unlike stressed cells, we have not detected induction of key biosynthetic genes along these pathways in three of the four AroG^*^ cell lines (Figure 8). Increased phenylpropanoid accumulation in these transformed lines was due most probably to increased production of the common substrates for these pathways, namely Phe and Tyr.

The two AAAs that increased significantly in the cells were Phe and Tyr (Figure 5), while Trp levels were below detection. Phe is considered the main AAA produced in plants under nonstressful conditions, with approximately 30% of the photosynthetic carbon directed through it to produce phenylpropanoids (Rippert and Matringe, 2002). Both Tyr and Trp levels are normally much lower than Phe in all plant systems studied. Here we found that Trp was undetectable, and the ratio between Phe and Tyr in control lines was much in favor of Phe (approximately 9:1), as in previous studies. However, in the AroG^*^ lines, the final concentration of Tyr was close to that of Phe, since the fold increase of Tyr was much higher than Phe (800 fold increase of Tyr compared to a 40 fold increase in Phe in line 13) (Figures 5C,D). This dramatic increase in Tyr concentrations in the grape AroG^*^ cell culture is unique in comparison to arabidopsis, petunia and tomato AroG^*^ lines, in which the effect of the transformation on Tyr concentrations was lower or similar to that of Phe (Tzin et al., 2012, 2013; Oliva et al., 2015). A possible explanation may be that Tyr plays a more significant role in the production of phenylpropanoids in the grape cell culture, and possibly in grape berries, than in arabidopsis leaves, tomato fruit or petunia flowers (Tzin et al., 2012, 2013; Oliva et al., 2015). Another possibility is that the catabolism of Tyr is slower in these cells.

**Figure 9:**
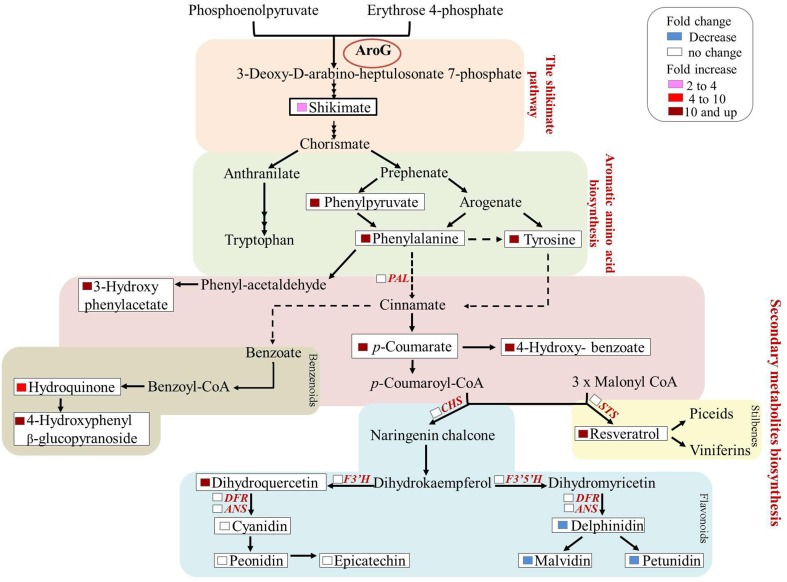
Summary of the metabolic and transcriptomic changes in the transgenic Gamay cell line with the highest AroG^*^ abundance, line 13. The scheme summarizes the fold change in the levels of AAA related metabolites in the AroG* lines in comparison to controls. In addition, the genes whose transcript levels were determined in line 13 are marked in red. None of these transcripts showed significant change.

Previous studies have shown that stress related induction of Phe and Tyr derived metabolites, namely phenylpropanoids, is due to induction of the Phe catabolic enzyme, PAL, as well as key enzymes in the specific specialized metabolic pathways, but not to induction of genes upstream of Phe and Tyr. This is in contrast to stress-related Trp-derived metabolites, that are overproduced due to induction of genes both along the biosynthetic pathway of Trp and those downstream of Trp (Figure 9, Less and Galili, 2008). There are only few reported cases in which induction of phenylpropanoids was accompanied by induction of the shikimate and AAA pathways in addition to those downstream of Phe (Ferrari et al., 2007). Here we show increased production of phenylpropanoid from several different pathways most probably due to the induction of flux through the shikimate pathway, suggesting that these pathways are “open” and the rate-limiting step is substrate availability (Figure 9). Increased production of phenylpropanoids due to increased levels of Phe has been reported recently in petunia flowers (Oliva et al., 2015). Both transformation of the plants with AroG^*^ and feeding non-transformed flower stems with labeled Phe resulted in an increase in volatile benzenoid phenylpropanoids (Oliva et al., 2015).

The effect of AroG^*^ overproduction both on the primary and specialized metabolites was in correlation to the abundance of AroG^*^ protein in four transformed lines, with the highest levels in line 13, which accumulated the highest concentration of AroG^*^ protein. The variations in the metabolic effects among the AroG^*^ lines were smaller here than in experiments with higher plants such as in petunia flowers (Oliva et al., 2015). This is expected due to the homogeneity of the cells, which were grown at constant and stable conditions. In this system the direct effect of AroG^*^ can be tested, without additional effects related to the whole plant organs and developmental stage. Since this grape cell culture can be viewed as a model for grape berries (Zhang et al., 2002), we assume that the metabolic effects observed here are close to what occurs in the fruit and thus may serve as a base for developing higher quality grapes.

The growth rate of the AroG^*^ cells was slightly slower than that of controls, due to energy shift toward phenylpropanoids biosynthesis. Similar decrease in the biomass of grape cell cultures was observed when cells were stressed by addition of jasmonic acid, heavy metals, growth regulators, and under drought stress (Williams et al., 2010; Cai et al., 2013, 2011). An additional difference in growth characteristics between AroG^*^ and control lines is that the AroG^*^ cells grew in larger clumps (Figure 3B). Plant cells in suspension cultures tend to form clumps. An increase in the size of the cell clumps has been suggested to correlate to phenylpropanoid production: When the anthocyanin pigmented strawberry cell culture FAR (*Fragaria ananassa* R) was treated with a PAL inhibitor, causing a decrease in the production of phenylpropanoids, the clumps formed during growth were much smaller compared to untreated cells producing anthocyanins and other phenylpropanoids (Edahiro and Seki, 2006). Increased clumping in the grape AroG^*^ cells may also be due to increased production of phenylpropanoids.

Total anthocyanin accumulation in line 13, with the highest accumulation of AroG^*^ protein, was identical to that in the control lines (Figure 4), resembling the effect of AroG^*^ on purple petunia flowers (Oliva et al., 2015). Anthocyanin accumulation in the two weaker lines, 50 and 51, that also grew at a slower rate than lines 3 and 13, was lower than controls and the cell cultures were lighter (Figures 3, 4). Interestingly, the flavonoid pathway up to dihydroquercetin is “open,” since this metabolite increased up to 150 fold in the AroG^*^ transformed lines. However, this dramatic increase in dihydroquercetin did not increase anthocyanin production. One explanation may be that the enzyme responsible for catabolism of dihydroquercetin, dihydroflavonol reductase (DFR), was not affected by increased substrate availability.

Even though total anthocyanin concentrations were not affected significantly by overproduction of Phe and Tyr, the ratios among the five anthocyanin aglycons changed, with significant decrease in delphinidin and delphinidin-derived aglycons (petunidin and malvidin) (Figure 7). Similar changes in the ratio between the grape anthocyanin aglycons were observed in grape cell cultures Gamay Freaux, when anthocyanin synthesis was induced due to treatments with sugar, jasmonic acid, exposure to light, etc. (Conn et al., 2010; Saw et al., 2012). This change suggests differential expression of the F3′H and F3′5′H genes or a change in the activity of their enzymes due to changes in the phenylpropanoid pathways. In the Gamay Red AroG^*^ cells, apart from line 3, there was no significant change in the expression of these two genes (Figure 8) suggesting that the control may be at the enzyme activity level.

Resveratrol, a phenylpropanoid considered to play a major role in the “French-paradox” phenomenon, increased over 20 fold in the strong AroG^*^ lines. A second metabolite that increased over 100 fold is dihydroquercetin, a precursor of quercetin. Both resveratrol and quercetin are considered health promoting metabolites due to their anti-oxidative characteristics and recent studies showing that both components induce so-called longevity genes (SIRT1-7), which protect humans from age-related diseases (Haigis and Sinclair, 2010). Our results suggest that the availability of Phe and Tyr is a rate limiting factor in the production of these metabolites in the Gamay cell culture. This raises an interesting question for further understanding how the production of these metabolites is controlled in grapes. What will be the effect of overexpressing specific genes directly producing these metabolites, such as STS and FLS for resveratrol and quercetin respectively, in AroG^*^ cells? Will induction of STS further increase resveratrol accumulation in cells in which substrate availability is not limiting?

### Conflict of interest statement

The authors declare that the research was conducted in the absence of any commercial or financial relationships that could be construed as a potential conflict of interest.
